# Inhibitory Effect of PgAFP and Protective Cultures on *Aspergillus parasiticus* Growth and Aflatoxins Production on Dry-Fermented Sausage and Cheese

**DOI:** 10.3390/microorganisms6030069

**Published:** 2018-07-13

**Authors:** Josué Delgado, Alicia Rodríguez, Alfredo García, Félix Núñez, Miguel A. Asensio

**Affiliations:** Food Hygiene and Safety, Institute of Meat Products, University of Extremadura, 10003 Cáceres, Spain; jdelgadoperon@gmail.com (J.D.); aliciarj@unex.es (A.R.); fredgarsa@gmail.com (A.G.); fnunez@unex.es (F.N.)

**Keywords:** Aflatoxin, *Aspergillus parasiticus*, antifungal protein, dry-fermented sausage, cheese, biopreservation, *Debaryomyces hansenii*

## Abstract

Aflatoxigenic molds can grow and produce aflatoxins on dry-fermented meat and cheese. The small, basic, cysteine-rich antifungal protein PgAFP displays a time-limited inhibitory ability against unwanted molds by increasing reactive oxygen species (ROS), which can lead to increased aflatoxin production. However, calcium abolishes the inhibitory effect of PgAFP on certain *Aspergillus* spp. To maximize the antifungal effect, this protein may be combined with protective cultures. Yeasts and lactic acid bacteria may counteract the impact of calcium on PgAFP fungal inhibition. The objective of this work was to study the effect of PgAFP and different combined treatments with *Debaryomyces hansenii* and/or *Pediococcus acidilactici* against growth of and aflatoxin production by an aflatoxigenic strain of *Aspergillus parasiticus* in both culture media and dry-fermented foods with low or high calcium levels. Aflatoxins production was increased by PgAFP but dramatically reduced by *P. acidilactici* in low calcium culture medium, whereas in the Ca-enriched culture medium, all treatments tested led to low aflatoxins levels. To study whether PgAFP and the protective microorganisms interfere with ROS and aflatoxin production, the relative expression of genes *foxA*, which is involved in peroxisomal β-oxidation, and *aflP*, which is required for aflatoxin biosynthesis, were evaluated. The aflatoxin overproduction induced by PgAFP seems not to be linked to peroxisomal β-oxidation. The combination of PgAFP and *D. hansenii* provided a successful inhibitory effect on *A. parasiticus* growth as well as on aflatoxin production on sliced dry-fermented sausage and cheese ripened up to 15 days, whereas *P. acidilactici* did not further enhance the protective effect of the two former agents. Therefore, the combined treatment of PgAFP and *D. hansenii* seems to provide a promising protective mean against aflatoxin-producing *A. parasiticus* on dry-fermented foods.

## 1. Introduction

Various microorganisms, including bacteria and fungi, decisively contribute to the specific characteristics of dry-fermented foods. However, the environmental conditions during the ripening of dry-fermented foods favor colonization of their surface by toxigenic molds that are able to produce mycotoxins on such foods. Aflatoxins have long been a major concern in cheese [1,2,3,4,5] and recent studies have highlighted their presence in dry-cured meats [6,7,8,9]. Aflatoxins, produced mainly by *Aspergillus flavus* and *Aspergillus parasiticus,* are classified as group 1 carcinogenic to humans by the International Agency for Research on Cancer. Therefore, it is necessary to design strategies to prevent aflatoxigenic molds growth on dry-fermented foods. The use of protective cultures could provide widely accepted preventive means to control mycotoxin production in ripened foods.

Some molds produce proteins that inhibit other molds and some yeasts, whereas the activity against prokaryotes is quite limited [10,11,12,13,14]. These antifungal proteins may serve to combat unwanted molds in foods. PgAFP from *Penicillium chrysogenum* [15] is one of these antifungal proteins with fungistatic effect, requiring less than 4.9 μg/mL for the highly sensitive molds, including *A. flavus* on dry-fermented sausage [16]. However, higher concentrations of up to 312.7 μg/mL are required for low sensitive molds, including *A. parasiticus*. In addition, the antifungal capability of this kind of proteins is dramatically reduced in presence of divalent cations, such as Ca^2+^ [17,18,19], rendering this protein ineffective against *A. flavus* on cheese [20]. PgAFP not only can inhibit growth, but also hamper aflatoxins biosynthesis. *A. flavus* treated with PgAFP for 24 h showed lower relative abundance of key enzymes in aflatoxin biosynthesis pathway, including O-methyltranferase, versicolorin B (VERB) synthase, and VER1 dehydrogenase [16]. However, the effect on aflatoxin production in low sensitive molds has not been tested yet. On the other hand, it is known that reactive oxygen species (ROS) contribute to aflatoxin biosynthesis [21,22], which makes it necessary to study the influence of subinhibitory concentrations of PgAFP on aflatoxin production.

The multifactorial mechanism of action of PgAFP increases ROS levels on sensitive fungal strains [16]. Some aspergilli are able to deal with the excess of toxic ROS by synthetizing aflatoxins, where peroxisomal β-oxidation of fatty acids plays a key role [23]. Expression of gene *foxA* has been used as a marker for β-oxidation in *A. flavus* [24] and *A. parasiticus* [25]. Similarly, the expression of gene *aflP*, coding for O-methyltransferase A, has been used to monitor aflatoxin biosynthesis [26,27]. Therefore, studying the expression of these two genes could provide information on whether PgAFP treatment increases aflatoxin production by oxidative stress.

On the other hand, the limited time extension of PgAFP antifungal activity makes it necessary to develop strategies that extend mold growth inhibition. A plausible alternative can be based on the combined use of PgAFP and microbial strains endowed with antifungal activity. Among the microorganisms usually present on dry-fermented products, yeasts thrive on dry-cured hams for the whole ripening time, being the dominant species *Debaryomyces hansenii* [28,29]. Some of these strains have shown antifungal capability against toxigenic *Penicillia* on dry-cured meat products [30,31,32]. Also, lactic acid bacteria have been proved as potent mold inhibitors [33]. *Pediococcus acidilactici* produces antimicrobial compounds [34,35], some of them being active against *A. parasiticus* [36].

The aim of this work was to investigate the antifungal capability of PgAFP alone and with *D. hansenii* and/or *Pediococcus acidilactici* against *A. parasiticus* in culture medium as well as on dry-fermented sausage and on cheese. To understand how these agents affect β-oxidation and aflatoxin production, their effect on *foxA* and *aflP* genes expression was also studied.

## 2. Material and Methods

### 2.1. Microbial Strains

Two mold strains obtained from the Spanish Type Culture Collection (CECT, Valencia, Spain) were used in this study: the PgAFP sensitive aflatoxin-producing *A. parasiticus* CECT 2682 [10] and the PgAFP-producer *P. chrysogenum* CECT 20922. In addition, the yeast *D. hansenii* FHSCC 253H from the microbial collection of Food Hygiene and Safety of the University of Extremadura (Cáceres, Spain) and *P. acidilactici* fargo 35 supplied by Laboratorios Amerex (Colmenar Viejo, Spain) were tested as potential antifungal organisms.

### 2.2. PgAFP Production and Purification

*P. chrysogenum* CECT 20922 was grown in potato dextrose broth (PDB; Scharlab, Barcelona, Spain) pH 4.5 incubated statically for 21 days at 25 °C. The mycelium was removed and the medium was filtered through a 0.22 μm-pore-size nylon membrane (MSI, Westboro, USA) to obtain a cell free medium. PgAFP was isolated from 450 mL of the cell-free medium through fast protein liquid chromatography (FPLC) with a cationic exchange column HiTrap SP HP (Amersham Biosciences, Uppsala, Sweden), further purified with a HiLoad 26/60 Superdex 75 gel filtration column (Amersham Biosciences) as previously described [37]. The isolated and concentrated protein was sterilized through filtration (0.22 µm, Thermo Fisher Scientific, Waltham, MA, USA) and its concentration was assessed by the Lowry method [38].

### 2.3. PgAFP and Microbial Inocula Preparation

*A. parasiticus* was grown on potato dextrose agar (PDA; Scharlab, Barcelona, Spain) for 15 days at 25 °C. Then, conidia were harvested by washing the surface of incubated plates with sterile phosphate-saline buffer (PBS). *D. hansenii* was grown in yeast extract sucrose (20 g/L yeast extract, 125 g/L sucrose; YES) broth for 72 h under continuous shaking at 200 rpm and 25 °C. YES medium containing *D. hansenii* was centrifuged to concentrate cells and the pellet was washed twice with sterile PBS. *P. acidilactici* was grown in de Man-Rogosa-Sharpe (MRS; Scharlab, Barcelona, Spain) broth for 24 h at 30 °C, and to concentrate the cells the same procedure previously described for yeasts was followed. Spore or yeast suspensions were counted in a Thoma counting chamber.

### 2.4. Mold Growth Inhibition and Mycotoxin Extraction in Culture Media

The effect of PgAFP, *D. hansenii,* and *P. acidilactici* on *A. parasiticus* was evaluated both separately and in different combinations. Samples were inoculated with *A. parasiticus* to reach 10^5^ conidia/mL or cm^2^. To provide a high level of potential antifungal agents, 10^6^ cells/mL or cm^2^ *D. hansenii*, 10^6^ cells/mL or cm^2^ *P. acidilactici*, or 10 µg/mL or cm^2^ PgAFP were added as required. An untreated batch inoculated only with *A. parasiticus* was used in each experiment as a control. The remaining batches were prepared using *A. parasiticus* and different combination of the aforementioned biopreservative agents, as described below.

The effect of PgAFP, *D. hansenii*, and *P. acidilactici* on *A. parasiticus* was evaluated in 5 mL YES broth and 0.1 M CaCl_2_-enriched YES (Ca-YES) broth. For this, the seven following treatments were tested in YES broth and Ca-YES broth: PgAFP alone (Pg), *D. hansenii* alone (Dh), *P. acidilactici* alone (Pa), PgAFP combined with *D. hansenii* (Pg + Dh), PgAFP combined with *P. acidilactici* (Pg + Pa), *D. hansenii* combined with *P. acidilactici* (Dh + Pa), and PgAFP combined with *D. hansenii* and *P. acidilactici* (Pg + Dh + Pa). Samples were incubated statically for 15 days at 25 °C to stimulate aflatoxin production [26]. All the assays were carried out in triplicate.

For mycotoxin extraction after incubation, test tubes were added with 5 mL of chloroform and shaken at 100 rpm for 1 h at room temperature in darkness. Then, the chloroform was separated and evaporated to dryness under a gentle stream of N_2_. To evaluate mold growth, the mycelium was recovered from the aqueous residue by filtering through a Miracloth (Calbiochem, Darmstadt, Germany), dried to constant weight at 100 °C, and weighed.

### 2.5. Mold Growth Inhibition on Dry-Ripened Foods

A commercial raw dry-fermented sausage (pH 5.8, 0.96 a_w_) shortly after stuffing in natural beef casing was cut into 7–8 mm thick slices. Similarly, commercial Gouda cheese was cut into 5 mm thick slices. Both sausage and cheese slices were dipped into ethanol to eliminate outer contamination. The ethanol was left to evaporate in a laminar flow cabinet Bio II (Telstar, Tarrasa, Spain) prior to placing the slices in ethanol-sterilized receptacles containing a saturated KCl solution to keep relative humidity constant at 84% after vapor-liquid equilibrium at 25 °C. *A. parasiticus* was inoculated on both sides of each sausage and cheese slice at 10^5^ conidia/cm^2^. No antifungal treatment was applied to control samples. The two treated batches were prepared by adding either 10 µg/cm^2^ PgAFP and 10^6^ cells/cm^2^ *D. hansenii* (Pg + Dh) or 10 µg/cm^2^ PgAFP and 10^6^ cells/cm^2^ each of *D. hansenii* and *P. acidilactici* (Pg + Dh + Pa) onto the sausage and cheese slices. All samples received the same total volume of liquid by adding the required amount of sterile phosphate-saline buffer (PBS). After spreading uniformly the inoculum over the cheese surface with a sterile bent glass rod, the liquid excess was left to dry in the laminar flow cabinet. Every batch was prepared in triplicate.

To stimulate aflatoxin production, all batches were incubated at 25 °C [26]. Samples were taken after 5 and 15 days of incubation. Water activity (a_w_) values were determined with a LabMaster-aw (Novasina, Lachen, Switzerland). *A. parasiticus* and *D. hansenii* counts were assessed on PDA incubated for 72 h at 25 °C, according to the characteristic colony morphology. Lactic acid bacteria counts were evaluated on MRS agar incubated for 72 h at 30 °C.

### 2.6. Mycotoxin Extraction

Mycotoxins produced on sausage or cheese slices were extracted as previously described [9]. Briefly, each sample slice was macerated under shaking in a dark flask with 60 mL acetonitrile-water (9:1, *v/v*) containing 0.1% formic acid, together with 50 mL hexane. The acetonitrile-water phase was recovered and filtered through sodium sulphate anhydrous. Then, the filtrate was mixed with additional 50 mL of hexane and shaken. The acetonitrile-water phase was filtered again and evaporated in a rotary evaporator at 40–45 °C. The residue was resuspended in 1 mL chloroform, filtered through a 0.45 µm nylon membrane (MSI, Westboro, MA, USA) and evaporated to dryness under a gentle stream of nitrogen.

### 2.7. Mycotoxin Quantification

The dried chloroform extracts from culture media, sausage, and cheese slices were resuspended in 100 μL of high-performance liquid chromatography (HPLC)-grade acetonitrile (Sharlab, Barcelona, Spain) and analyzed by ultra high-performance liquid chromatography–mass spectrometry (UHPLC-MS) in a Dionex UltiMate 3000 HPLC system (Thermo Fisher Scientific) coupled to an ion trap mass spectrometer (MS) model Amazon SL (Bruker Daltonics, Bremen, Germany). A C18 reverse-phase column of 10 cm length, 2.1 mm inner diameter and 1.8 μm particle size (Agilent Technologies, Santa Clara, CA, USA) was used as stationary phase. The mobile phases were (A) 0.1% formic acid-10 mM ammonium formate and (B) acetonitrile. The separation was performed at 200 µL/min flow rate and the following gradient: 0 min, 2% B; 0–0.1 min, 2–40% B; 0.1–4 min, 40–60% B; 4–7 min, 60–80% B; 7–8.5 min, 80% B; 8.5–8.51 min, 80–98% B; 8.51–12 min, 98% B; 12–12.01 min, 98–2% B; and 12.01–15 min, 2% B. Retention time for aflatoxins B_1_ and G_1_ (AFB_1_ and AFG_1_) were 6.4 ± 0.5 and 6.3±0.5 min respectively. Precursor ions 313 and 329, and quantitation ions 285 and 311 for AFB_1_ and AFG_1_, respectively, were used.

These parameters were compared to those obtained from commercial mycotoxins (Sigma-Aldrich, Madrid, Spain). The calibration curves for AFB_1_ and AFG_1_ (1–500 ng) by uHPLC-MS revealed a linear relationship (r^2^ ≥ 0.99) between the detector response and the amount of AFB_1_ and AFG_1_ standards. The minimum detectable value or limit of detection (LOD) was estimated from the calibration curve, according to the equation: LOD = 3 (s_B_^2^ + s_i_^2^ + (i/m)^2^ s_m_)^1/2^/m [39], with “m” being the slope of the calibration curve, “I” being the intercept term, and “s_B_”, “s_i_”, and “s_m_” being the standard errors of the blank, the intercept term, and the slope of the calibration curve, respectively. Assuming a normal distribution of the estimated quantities, α (error of the first type) = β (error of the second type) = 0.05, the quantification limit (LOQ) was 3.04 LOD [40]. The LOD obtained in this study were 4 ppb and 1.5 ppb, and the LOQ were 12 ppb and 4.5 ppb, respectively, for AFB_1_ and AFG_1_.

### 2.8. Gene Expression Studies

To assess the influence of PgAFP alone or in combination with *D. hansenii* and *P. acidilactici* on *A. parasiticus* gene expression, four batches were prepared in YES broth and Ca-YES broth. *A. parasiticus* was co-cultivated in tubes with Pg, Dh, Pa, and Pg + Dh + Pa. An untreated batch was inoculated solely with *A. parasiticus* (control). The tubes were incubated at 25 °C for 5 days. After this, mycelium was harvested and washed twice with sterile PBS, frozen in liquid nitrogen, ground with a mortar and pestle, and stored at −80 °C until RNA extraction.

#### 2.8.1. RNA Isolation and Complementary DNA Synthesis

For RNA extraction, the frozen ground mycelium was resuspended in 750 µL RLT buffer (RNeasy^®^ Plant Mini kit, Qiagen, Hilden, Germany) containing 15 µL β-mercaptoethanol. Samples were processed with the RNeasy^®^ Plant Mini kit according to manufacturer’s instructions. RNA quality and quantity were spectrophotometrically determined using in a NanoDrop 2000c spectrophotometer (Thermo Scientific, Waltham, MA, USA). To remove genomic DNA contamination, samples were treated with DNase I, RNase-free (Fermentas, St. Leon-Rot, Germany) following manufacturer’s instructions. Finally, complementary DNA (cDNA) was synthesized using 500 ng of total RNA according to the PrimeScript™ RT Reagent kit protocol (Takara, Otsu, Japan).

#### 2.8.2. Relative Quantification of Gene Expression by Real Time-Polymerase Chain Reaction

The expression of genes *aflP* (formerly *omt-1*) for aflatoxin biosynthesis [27], *foxA* for β-oxidation [25], and *β-tubulin* as housekeeping marker [41] were assessed to evaluate the effect of the different treatments on *A. parasiticus*. Primers F-Omt-1 and R-Omt-1, previously designed by [42], were used to evaluate *aflP* expression. To evaluate *foxA* expression, primers F-foxA and R-foxA were designed from the gene AFLA_041590 (GenBank accession no. XM_002377580). Similarly, to study *β-tubulin* expression, primers F-β-tub and R-β-tub were designed from *A. parasiticus β-tubulin* sequence (GenBank accession no. FR775333.1). Primers were designed on exon in the target using the Primer-Blast tool from the National Center for Biotechnology Information (NCBI) (http://www.ncbi.nlm.nih.gov/tools/primer-blast/). Nucleotide sequences of primers used in the real time-quantitative polymerase chain reaction (RT-qPCR) assays are shown in Table 1.

The ViiATM 7 Real-Time PCR System (Applied Biosystems, Foster City, CA, USA) was used to carry out the RT-qPCR assays. To optimize the primer concentration, different concentrations from 800 to 200 nM were tested. SYBR Green methodology was applied. The optimized SYBR Green protocols were carried out in a final volume of 25 µL containing 5 µL of template cDNA, 12.5 µL of 2x SYBR^®^ *Premix Ex Taq*™ (Takara, Otsu, Japan), 0.5 µL of 50x ROX™ Reference Dye (Takara) and 400 nM of each primer. The thermal cycling conditions were the following: a single step of 10 min at 95 °C, 40 cycles of 95 °C for 15 s, and 60 °C for 1 min. After the final PCR cycle, melting curves analysis of the PCR products were carried out by heating from 60 to 95 °C and continuous measurement of the fluorescence to verify the PCR product. The *aflP* gene amplification was evaluated following the concentrations and conditions previously described [42]. Ct determinations were automatically performed by the instrument using defaults parameters. For every primer pair, a standard curve was generated to check the amplification efficiency from ten-fold serial dilution 100 to 0.01 ng/µL of DNA from *A. parasiticus*. Every qPCR assay described above was carried out by triplicate.

To quantify the relative expression of the genes, *foxA* and *β-tubulin* genes, the 2^−ΔΔCT^ method was used [43]. The endogenous control was set as the expression of *β-tubulin* gene and the untreated samples (control batch) was used as calibrator sample. To check the amplification efficiency for every primer pair, a standard curve was generated from 10-fold serial dilutions (100 to 0.01 ng/µL) of DNA from *A. parasiticus*. The differences in amplification efficiencies between the housekeeping and the target gene were 3 and 5% for *aflP* and *foxA* genes, respectively, as required to apply the relative quantification method [44].

### 2.9. Statistical Analysis

Statistical analyses were performed with the IBM SPSS v.22. Data from mycelia weight, microbial counts, mycotoxin concentration and gene expression were tested for normality (Kolmogorov-Smirnov with Lilliefors correction) and homoscedasticity (Levene’s test). Given that these data were non-normally distributed, untreated and treated samples were compared in pairs using the nonparametric Mann-Whitney U test (*p* ≤ 0.05). To study the relationship between expression of *aflP* and *foxA* genes, a Spearman correlation was applied (*p* ≤ 0.05). 

## 3. Results

### 3.1. A. parasiticus Growth Inhibition and Mycotoxin Production in Culture Media

**Figure 1:**
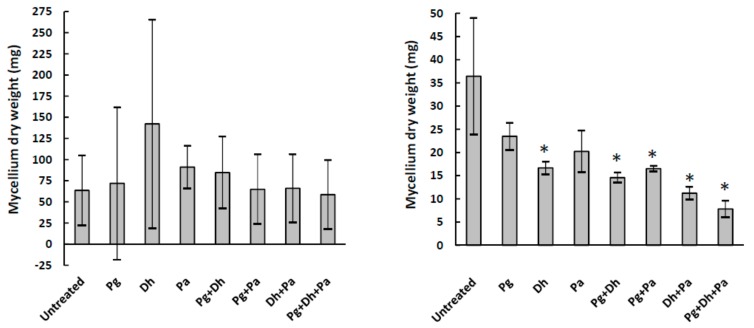
Mycelium dry weight of *A. parasiticus* cultured in yeast extract sucrose broth (left) and calcium-enriched yeast sucrose broth (right) for 15 days with different combinations of biopreservative agents. Pg: PgAFP, Dh: *Debaryomyces hansenii*, Pa: *Pediococcus acidilactici.* * Means are significantly different from untreated control (*p* ≤ 0.05).

To study the antifungal capability of the three agents, both separately and in different combinations, seven treatments (Pg, Dh, Pa, Pg + Dh, Pg + Pa, Dh+Pa, and Pg + Dh + Pa) were tested against *A. parasiticus* for 15 days in YES and Ca-YES broth. *A. parasiticus* growth was substantial in YES broth, regardless of the treatment applied (Figure 1). As a result of the high standard deviation, there was no statistically significant difference (*p* > 0.05) in *A. parasiticus* growth between any treatment and the untreated control. On the other hand, *A. parasiticus* grew poorly in Ca-YES broth and was further inhibited by most treatments, but not by PgAFP alone (Figure 1).

**Figure 2:**
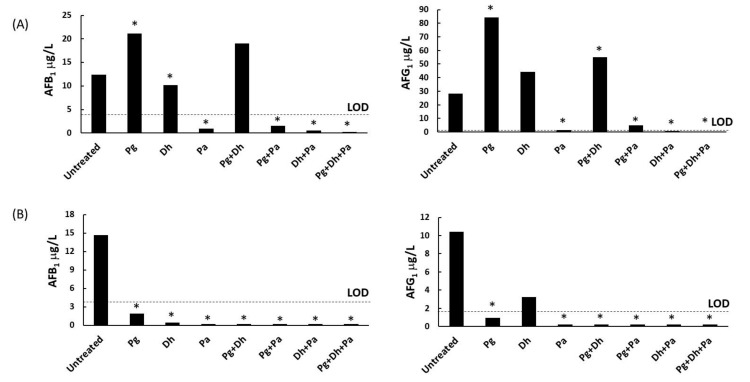
Effect of different combinations of biopreservative agents in aflatoxin B_1_ (left) and G_1_ (right) production by *Aspergillus parasiticus* in yeast extract sucrose broth (**A**) and calcium-enriched yeast extract sucrose broth (**B**) after 15 days. Pg: PgAFP; Dh: *Debaryomyces hansenii*; Pa: *Pediococcus acidilactici*. LOD: Limit of detection. * Means are significantly different from untreated batch (*p* ≤ 0.05).

Both AFB_1_ and AFG_1_ were produced in the untreated batch by *A. parasiticus* in both culture media (Figure 2). Aflatoxin production showed differences (*p* ≤ 0.05) depending on the treatment applied. PgAFP treatment increased AFB_1_ and AFG_1_ production in YES broth, but led to lower aflatoxin concentrations in Ca-YES. All treatments containing *P. acidilactici* strongly inhibited aflatoxins production. Batches inoculated with *D. hansenii* also showed lower AFB_1_ production in both culture media. In YES broth, Pg + Dh induced a higher AFG_1_ quantity whereas no statistically significant increase was obtained for AFB_1_ compared to the untreated control. In Ca-YES broth, *A. parasiticus* produced both AFB_1_ and AFG_1_ above the LOD only in the untreated control, and AFG_1_ in the batch treated solely with *D. hansenii*. Therefore, PgAFP neither alone nor combined with any of the remaining protective agents tested increased aflatoxin production in Ca-YES broth.

### 3.2. A parasiticus Growth Inhibition and Mycotoxin Production on Sliced Sausage

The effect of PgAFP-combined treatments on mold development was evaluated on slices of raw sausage kept under controlled environmental conditions simulating normal ripening. A_w_ values reached 0.93 and 0.86 at 5 and 15 days of incubation, respectively (Table 2). The microbial load in the pre-sterilized sliced sausage just before inoculation was 2.3 log cfu/cm^2^ for molds, 1 log cfu/cm^2^ for lactic acid bacteria, and lower than 1.7 log cfu/cm^2^ for yeasts. After treatment application, yeast counts were always over 5 log cfu/cm^2^ in *D. hansenii-*inoculated batches, but lower than 2 log cfu/cm^2^ in the untreated batch. Similarly, MRS counts were over 6.8 log cfu/cm^2^ in *P. acidilactici*-inoculated samples, but lower than 4.5 log cfu/cm^2^ in the remaining ones. Mold counts from untreated batches were over 6.5 and 7.4 log cfu/cm^2^ at five and 15 days, respectively (Table 2). Pg + Dh or Pg + Dh + Pa treatments dramatically decreased *A. parasiticus* counts by about 3 log units.

On the other hand, mycotoxin production in the sausages was somehow parallel to fungal growth. *A. parasiticus* produced high AFB_1_ and AFG_1_ levels in untreated sausages, both at five and 15 days of incubation (Table 2). However, aflatoxin production in Pg + Dh and Pg + Dh + Pa batches was lower (*p* ≤ 0.05) than in the untreated control at both sampling times.

### 3.3. A. parasiticus Growth Inhibition and Mycotoxin Production on Cheese Slices

The microbial load on cheese slices just before inoculation was about 3 log cfu/cm^2^ for lactic acid bacteria (LAB) and below 2 log cfu/cm^2^ for both molds and yeasts (Table 3). LAB increased just after inoculation to 6–6.7 log cfu/cm^2^ in Pg + Dh + Pa batch, which was the only group of cheese samples inoculated with *P. acidilactici*. At five and 15 incubation days, LAB counts remained around 3.5 log cfu/cm^2^ for batches not inoculated with *P. acidilactici* (untreated and Pg + Dh), but over 6 log cfu/cm^2^ for Pg + Dh + Pa batches. Similarly, the yeast load was always below 2 log cfu/cm^2^ in the untreated control, but over 5 log cfu/cm^2^ in Pg + Dh and Pg + Dh + Pa batches. The a_w_ value of cheese slices after inoculation was 0.96, and went down to 0.86 at 15 days of incubation (Table 3). Fungal loads at five days were around 2 log units higher (*p*≤0.05) in untreated than in Pg + Dh and Pg + Dh + Pa batches (Table 3). At 15 days, fungal counts in untreated samples were lower than 5 log cfu/cm^2^. Counts around 2–3 log cfu units lower (*p* ≤ 0.05) were also obtained with Pg + Dh and Pg + Dh + Pa treatments after incubation for 15 days.

The only mycotoxin obtained from cheese at the conditions tested was AFG_1_ produced by *A. parasiticus* in the untreated batch at 5 and 15 days (Table 3). The high AFG_1_ levels detected in these samples at day 15 were well over the 10–20 μg/kg limit established for some foods in different countries.

### 3.4. Gene Expression

**Figure 3:**
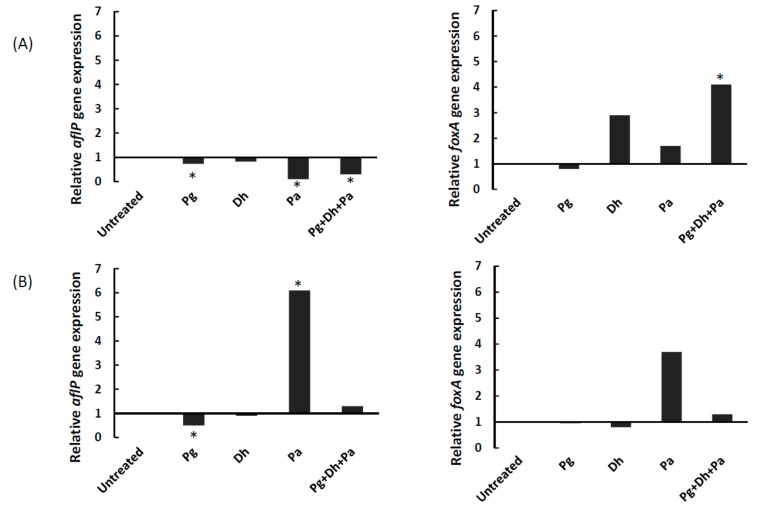
Effect of different combinations of biopreservative agents on relative gene expression of *aflP* (left) and *foxA* (right) genes in yeast extract sucrose broth (**A**) or calcium-enriched yeast extract sucrose broth (**B**) after five days. Pg: PgAFP; Dh: *Debaryomyces hansenii*; Pa: *Pediococcus acidilactici.* *Means are significantly different from untreated batch (*p* ≤ 0.05).

The effect of PgAFP, *D. hansenii*, and *P. acidilactici* on the expression of genes *aflP* for aflatoxin biosynthesis, *foxA* for β-oxidation, and *β-tubulin* as housekeeping were assessed in *A. parasiticus* cultures grown in YES and Ca-YES broth for five days, when PgAFP activity on *A. parasiticus* is expected to be high. The expression of *aflP* gene was repressed in *A. parasiticus* by the sole action of either PgAFP or *P. acidilactici* as well as the Pg + Dh + Pa combined treatment in YES broth (Figure 3). When *A. parasiticus* was grown in Ca-YES broth, *aflP* was also repressed (*p* ≤ 0.05) in Pg batch, but overexpressed (*p* ≤ 0.05) in Pa batch (Figure 3).

The expression of *foxA* only differed (*p* ≤ 0.05) from its untreated control in the Pg + Dh + Pa samples from YES broth, leading to a 4-fold overexpression (Figure 3). No treatment led to statistically significant changes for *foxA* expression in Ca-YES broth, despite the high expression rate obtained from Pa samples (Figure 3). In addition, *foxA* gene expression values did not correlate with a*flP* expression values (*p* > 0.05).

## 4. Discussion

As expected, PgAFP did not inhibit *A. parasiticus* growth in YES or Ca-YES media (Figure 1). The level of 10 µg/mL PgAFP used was too low to reach a significant effect, as it was shown previously [10]. However, to test the effect of PgAFP on aflatoxins production, a concentration below that causing moderate inhibition had to be tested. Additionally, neither *P. acidilactici* nor *D. hansenii* effectively inhibited *A. parasiticus* growth in the YES medium. On the other hand, most treatments tested inhibited (*p* ≤ 0.05) *A. parasiticus* growth in Ca-YES broth (Figure 1). The highest inhibition reached by Pg + Dh + Pa seemed to be a consequence of the combined effect of the different mechanisms of action. PgAFP activity is based on permeability induction, loss of membrane integrity, and apoptosis induction as a consequence of ROS [16], whereas the inhibitory effect of *D. hansenii* is attributed to volatile compounds and competition for nutrients and space [31]. In addition, *P. acidilactici* produces bacteriocins and organic acids that inhibit mold growth [35,45].

Pg + Dh and Pg + Dh + Pa treatments showed a remarkable inhibition on *A. parasiticus* on the sliced sausages (Table 2). Mold counts in non-treated sausages were higher than those in Pg + Dh and Pg + Dh + Pa batches by Day 5. Given that 3.5 log cfu/cm^2^ has been suggested as the limit to keep minimal risk of AFB_1_ accumulation in dry-cured hams [9], the two PgAFP-combined treatments applied could be useful to control aflatoxigenic molds in dry-cured meat products. At 15 days incubation time, which covers most of the ripening stage of dry-fermented sausages, mold counts in treated samples were still around 0.1% of the mold load on the untreated samples.

The lack of inhibitory effect of the tested antifungal agents in the culture medium as compared to the efficient inhibition in dry-fermented sausages can be attributed to the differences in substrate availability. The mode of action proposed for yeasts to control molds in culture media is competition for nitrogen compounds, sugars, and vitamins [46]. In addition, some volatile compounds derived from branched amino acids by *D. hansenii* are responsible for mold inhibition [31,47]. YES broth contains 125 g/L sucrose and 20 g/L yeast extract, providing readily available sources of carbon and nitrogen compounds, as well as vitamins, especially from the B complex. Conversely, sausages contain lower amounts of sugars and vitamins, but higher levels of free amino acids throughout the ripening process [48,49]. Therefore, these differences could be the key factor for the efficient inhibition of *A. parasiticus* by *D. hansenii*, and *P. acidilactici* combined with PgAFP on sliced sausage.

On the other hand, the same strain of *D. hansenii* used here was barely active at low (0.84) compared to high (0.94) a_w_ values when grown on dry-cured ham slices for 15 days [30]. Therefore, *D. hansenii* alone might not efficiently inhibit *A. parasiticus* on sausages at a_w_ values around 0.85. Conversely, the combination of PgAFP and *D. hansenii* (Pg + Dh) efficiently inhibited *A. parasiticus* growth on sliced sausage (Table 2). Thus, the combined PgAFP could be decisive to help the other biological agents reach an efficient inhibition of mycotoxigenic molds in dry-cured meats, particularly at low a_w_ values.

The impact of PgAFP on aflatoxins production greatly differs with the culture medium used (Figure 2). The increased aflatoxins levels in YES broth is attributed to ROS induction by antifungal proteins, which is a prerequisite for aflatoxin production in *A. parasiticus* [21]. Conversely, the decreased aflatoxins levels in Ca-YES broth must be related to the calcium-triggered resistance against PgAFP mediated by calcineurin, G protein, and γ-glutamyltraspeptidase, that combat oxidative stress in *A. flavus* [20]. In fact, aflatoxin production was also inhibited in Ca-YES by most of the tested treatments, except for AFG_1_ in the batch inoculated only with *D. hansenii* (Figure 2).

Cocultivation of *A. parasiticus* with *P. acidilactici* in YES led to lower AFB_1_ production, irrespective of the addition of PgAFP (Figure 2). Therefore, *P. acidilactici* seems to prevent the increment of mycotoxin production provoked by PgAFP. The mechanism behind the lower aflatoxin production with lactic acid bacteria is not known, but it has been related to low molecular weight inhibitory compounds as well as removing due to aflatoxin binding [50].

The oxidative status in the fungal cell severely affects mycotoxin production [51]. Several antifungal proteins from molds provoke ROS generation in sensitive strains [18,19], including PgAFP in *A. flavus* [16]. ROS production is regarded as a prerequisite for the onset of aflatoxin production [22,23]. Conversely, several antioxidant substances and enzymes inhibit aflatoxin production [22,52]. For all this, the effect of PgAFP, used alone or combined with the two microbial agents, on the expression of *foxA* and *aflP* genes was studied in *A. parasiticus*.

ROS induction has been proposed to play a key role in the mechanism of action of PgAFP on sensitive molds [16]. The effect of ROS on aflatoxin synthesis has been related to redox balance through β-oxidation, as studied through *foxA* gene expression [23]. Besides, the *aflP* gene has been used to study the effect of environmental factors on aflatoxin production [26]. Thus, *aflP* and *foxA* expression was assessed to study the effect of PgAFP and the other two biocontrol agents on aflatoxin production.

*aflP* expression in *A. parasiticus* grown in YES broth was not overexpressed, but repressed by PgAFP, as well as by *P. acidilactici* and the combined Pg + Dh + Pa treatment (Figure 3). In Ca-YES broth, *aflP* expression was also repressed by PgAFP, but overexpressed in samples treated only with *P. acidilactici*. Despite the differences in *aflP* expression, no statistically significant difference was obtained in the aflatoxin levels from these batches. The lack of correlation between gene expression and aflatoxin production may be related to the fact that *aflP* is a structural rather than a regulatory gene [53]. According to these results, the potential effect of PgAFP promoting aflatoxin production has to be ruled out in calcium-enriched media.

On the other hand, the *foxA* gene expression showed no statistically significant difference due to PgAFP treatment in both YES and Ca-YES broth. These results are consistent with the lower relative abundance of O-methyltransferase, coded by the *aflP* gene, and the unaffected level of peroxisomal multifunctional β-oxidation protein, coded by the *foxA* gene, found in PgAFP-treated *A. flavus* [16], as well as with the low levels of intracellular ROS detected in PgAFP-treated *A. flavus* grown in Ca-enriched PDB [20]. In addition, *D. hansenii* did not alter *foxA* expression, supporting that the effect of this yeast reducing aflatoxin levels is not due to a potential antioxidant effect. Therefore, the overproduction of aflatoxin observed in PgAFP-treated *A. parasiticus* grown in Ca-YES broth (Figure 2) may not be initiated by an increased β-oxidation due to ROS. In addition, the increased *foxA* expression obtained in Pg + Dh + Pa treated samples from YES broth was not accompanied by higher aflatoxin production at the 15th day. These results suggest that the effect of ROS on aflatoxin biosynthesis cannot be explained solely through β-oxidation. The generation of secondary ROS in endosomes upon exposure to exogenous ROS during aflatoxins biosynthesis [54] suggests that other pathways may be involved in the metabolism of these toxic compounds, such as calcineurin signaling related to the role of calcium as a secondary messenger in the response to antifungal proteins in fungi [55]. However, these studies are still in a preliminary stage and require further investigation.

*A. parasiticus* produced AFB_1_ and AFG_1_ when grown on the untreated sausages, reaching levels well above the levels regarded as safe for various foods [56] both at five and 15 days of incubation (Table 1). The amount of AFB_1_ and AFG_1_ produced on sausages was higher than that obtained in YES broth, which can be explained by the limited availability of nutrients [57] and the increased levels of free amino acids [58] in the sausage. Aflatoxin production was greatly reduced by Pg + Dh and Pg + Dh + Pa treatments on sausage samples (Table 2). AFB_1_ and AFG_1_ were not detected in treated samples at Day 5 of incubation and only slightly above the limits set for foods [56] at 15 days. However, given that the conditions set were favorable for *A. parasiticus* development and mycotoxin production, it is expected that any of the two combinations tested may be sufficient to control the usual fungal contamination during dry sausage ripening. 

The successful antifungal effect of both treatments with Pg + Dh on dry-fermented sausage supports their use as a preventive measure against aflatoxigenic *A. parasiticus* or as a corrective action when this mold has been detected on the meat product. The inclusion of *P. acidilactici* as an additional protective culture did not substantially improve the inhibition on *A. parasiticus* growth or mycotoxin production. Thus, the contribution of *P. acidilactici* seems to be negligible. This fact has to be related to the poor ability of this bacterium to grow on dry-fermented sausages.

Given that both the mycelial weight and mycotoxin production were inhibited by some treatments in Ca-YES broth, the efficacy of the most effective treatments containing PgAFP was tested on cheese slices. The low a_w_ levels reached during cheese ripening, even lower than 0.90 in the rind of some cheeses [59], could restrict survival of *P. acidilactici* and *D. hansenii*. The a_w_ values in treated cheese slices decreased to 0.89 and 0.86 at Days 5 and 15, respectively (Table 2), which are below the 0.90 minimum a_w_ level for *Pediococcus* growth in foods [60] , but not below the 0.83 minimum value for *D. hansenii* [61] and aflatoxins formation by *A. parasiticus* [62].

On cheese slices, both Pg + Dh and Pg + Dh + Pa treatments effectively inhibited *A. parasiticus* growth and aflatoxins production for 15 days. This effect was stronger than that reported by the sole action of *D. hansenii* on ochratoxigenic *P. nordicum* in dry-cured ham incubated at 0.84 a_w_ [30]. No additional inhibition was observed by the combined use of *P. acidilactici* with PgAFP and *D. hansenii* (Table 2), which could be due to *P. acidilactici* failing to thrive under the restrictive a_w_ values. However, the samples inoculated with *P. acidilactici* showed the highest LAB load. Although production of antifungal proteinaceous compounds by LAB in fresh cheese has been reported [63], the lower a_w_ values in ripened cheese might limit the production of antifungal compounds by *P. acidilactici*.

In conclusion, the combined treatment of PgAFP and *D. hansenii* inhibits *A. parasiticus* growth and mycotoxin production in dry-fermented sausages and cheese at similar conditions to those during the ripening process. *P. acidilactici* down-regulates *aflP* gene in *A. parasiticus* grown in YES broth, lowering aflatoxin production, but it does not provide further protection in sausage or cheese treated with PgAFP and *D. hansenii*. The increased aflatoxin production induced by PgAFP does not involve an increased *foxA* expression, but knowing the exact role of ROS will require further studies. On the whole, these results confirm the usefulness of PgAFP to control the aflatoxigenic population on dry-fermented foods, even on calcium-rich ones.

## Figures and Tables

**Table 1:** Nucleotide sequence of primers used for the quantitative polymerase chain reaction (qPCR) assay.

Primer Name	Nucleotide Sequences (5′-3′)	Product Size	Position	Reference
F-Omt-1	GGCCGCCGCTTTGATCTAGG	123	^a^ 1485	[42]
R-Omt-1	ACCACGACCGCCGCC		1593	
F-foxA	ACCGCAACCCTCTTCACATT	73	^b^ 1196	This study
R-foxA	AGACCGTGCAGGATAGGAGT		1268	
F-β-tub	TCTTCATGGTTGGCTTCGCT	98	^c^ 964s	This study
R-β-tub	CTTGGGGTCGAACATCTGCT		1042	

^a^ Positions are in accordance with the published sequence of the *aflP* (*omt-1*) gene of *A. flavus* (GenBank accession no. L25835.1). ^b^ Positions are in accordance with the published sequence of the *foxA* gene of *A. flavus* (GenBank accession no. XM_002377580). ^c^ Positions are in accordance with the published sequences of the *β-tubulin* gene of *A. parasiticus* (GenBank accession no. FR775333.1).

**Table 2:** Aflatoxins concentrations and microbial counts on slices of dry-fermented sausage at five and 15 days of incubation. Data are given as mean ± SD.

	Day 5		
	Untreated	Pg + Dh *^a^*	Pg + Dh + Pa
**Mold counts** (log cfu/cm^2^)	6.54 ± 0.22	<3 *	<3 *
**Yeast counts** (log cfu/cm^2^)	<3.00	>7.50 *	>7.50 *
**Lactic acid bacteria** (log cfu/cm^2^)	4.40	3.54	7.53 *
**Aflatoxin B_1_** (μg/kg)	86 ± 8	<LOD ^b^^,^*	<LOD *
**Aflatoxin G_1_** (μg/kg)	152 ± 9	<LOD *	<LOD *
	**Day 15**		
	**Untreated**	**Pg + Dh**	**Pg + Dh + Pa**
**Mold counts** (log cfu/cm^2^)	7.42 ± 0.09	4.26 ± 0.28 *	4.74 ± 0.38 *
**Yeast counts** (log cfu/cm^2^)	<3.00	>7.50 *	6.05 ± 0.48 *
**Lactic acid bacteria** (log cfu/cm^2^)	4.30	3.23	6.83 *
**Aflatoxin B_1_** (μg/kg)	115 ± 0.43	<LOD *	<LOD *
**Aflatoxin G_1_** (μg/kg)	170 ± 31	8.3 ± 4.5 *	10 ± 1.3 *

*^a^* Pg: PgAFP; Dh: *Debaryomyces hansenii*; Pa: *Pediococcus acidilactici. ^b^* LOD: Limit of detection. * Mean values are significantly different from those of the untreated batch (*p* ≤ 0.05).

**Table 3:** Water activity (a_w_), microbial counts and aflatoxins concentration in cheese slices inoculated with *A. parasiticus *at five and 15 days of incubation. Data are given as mean ± SD.

	Day 5			
	Untreated		Pg + Dh *^a^*	Pg + Dh + Pa
**A_w_**	0.89		0.89	0.89
**Mold count** (log cfu/cm^2^)	4.12 ± 0.57		2.31 ± 0.64 *	2.39 ± 0.75 *
**Yeast count** (log cfu/cm^2^)	<3.00		>7.50 *	>7.50 *
**Lactic acid bacteria** (log cfu/cm^2^)	2.84 ± 0.35		2.49±0.74	6.73 ± 0.26 *
**Aflatoxin B_1_** (µg/kg)	<LOD *^b^*		<LOD	<LOD
**Aflatoxin G_1 _**(µg/kg)	11 ± 7		<LOD *	<LOD *
	**Day 15**			
	**Untreated**	**Pg + Dh**	**Pg + Dh + Pa**	
**A_w_**	0.86	0.86	0.86	
**Mold count** (log cfu/cm^2^)	4.87 ± 0.21	<2 *	2.99 ± 0.67 *	
**Yeast count** (log cfu/cm^2^)	<3.00	>7.50 *	>7.50 *	
**Lactic acid bacteria** (log cfu/cm^2^)	3.75	2.7	6.71 *	
**Aflatoxin B_1_** (µg/kg)	<LOD	<LOD	<LOD	
**Aflatoxin G_1_** (µg/kg)	29 ± 24	<LOD	<LOD	

*^a^* Pg: PgAFP; Dh: *Debaryomyces hansenii*; Pa: *Pediococcus acidilactici. ^b^* LOD: Limit of detection. * Means are significantly different from untreated samples (*p* ≤ 0.05).
